# Functional Identification of Dendritic Cells in the Teleost Model, Rainbow Trout (*Oncorhynchus mykiss*)

**DOI:** 10.1371/journal.pone.0033196

**Published:** 2012-03-12

**Authors:** Elizabeth Bassity, Theodore G. Clark

**Affiliations:** Department of Microbiology and Immunology, College of Veterinary Medicine, Cornell University, Ithaca, New York, United States of America; INRA, France

## Abstract

Dendritic cells are specialized antigen presenting cells that bridge innate and adaptive immunity in mammals. This link between the ancient innate immune system and the more evolutionarily recent adaptive immune system is of particular interest in fish, the oldest vertebrates to have both innate and adaptive immunity. It is unknown whether dendritic cells co-evolved with the adaptive response, or if the connection between innate and adaptive immunity relied on a fundamentally different cell type early in evolution. We approached this question using the teleost model organism, rainbow trout (*Oncorhynchus mykiss*), with the aim of identifying dendritic cells based on their ability to stimulate naïve T cells. Adapting mammalian protocols for the generation of dendritic cells, we established a method of culturing highly motile, non-adherent cells from trout hematopoietic tissue that had irregular membrane processes and expressed surface MHCII. When side-by-side mixed leukocyte reactions were performed, these cells stimulated greater proliferation than B cells or macrophages, demonstrating their specialized ability to present antigen and therefore their functional homology to mammalian dendritic cells. Trout dendritic cells were then further analyzed to determine if they exhibited other features of mammalian dendritic cells. Trout dendritic cells were found to have many of the hallmarks of mammalian DCs including tree-like morphology, the expression of dendritic cell markers, the ability to phagocytose small particles, activation by toll-like receptor-ligands, and the ability to migrate *in vivo*. As in mammals, trout dendritic cells could be isolated directly from the spleen, or larger numbers could be derived from hematopoietic tissue and peripheral blood mononuclear cells *in vitro*.

## Introduction

While dendritic cells (DCs) are named for their distinctive morphology, their efficacy in stimulating naïve T cell responses distinguishes them from the ranks of other antigen presenting cells (APCs) [1], [2]. Their superior ability to stimulate T cell division results from their unique activation process which is driven by contact with pathogen- or damage-associated molecular patterns (PAMPs, or DAMPs) through pattern recognition receptors [3]. Activation involves uptake of antigen, production of cytokines, and migration to secondary lymphoid tissue. En route to spleen and lymph nodes, DCs upregulate surface MHC complexed with processed antigen peptides, CD83, and costimulatory molecules, concurrent with the loss of their phagocytic capacity [4], [5]. Besides the physical transport, processing, and presentation of antigen, the ligation of DC PAMP and DAMP receptors at the site of infection or inflammation informs their control of the type of adaptive response initiated [6].

The existence of mammalian DCs was long overlooked due to their rarity *in vivo* and behind-the-scenes orchestration of the immune response. First isolated from mouse spleen in the early 1970's, DCs compose a maximum of 1.6% of nucleated cells in this tissue [1]. Subsequently, methods were devised of culturing larger numbers of cells *in vitro* from bone marrow [7] and peripheral blood monocytes [8], enabling extensive characterization of DCs.

Although mammalian DCs (mDCs) have undergone intense scrutiny in recent years, questions regarding how and when these cells evolved remain unaddressed. Jawed fishes are the earliest vertebrates capable of adaptive immunity (involving MHC, BCR and TCR), and the molecular machinery necessary for antigen processing and presentation is present and functional in these species [9], [10], [11], [12]. There is significant evidence that mounting of adaptive immune responses occurs in much the same way in jawed fish as in mammals [13]. Therefore, it stands to reason that a specialized cell type that connects innate and adaptive immunity through antigen presentation is present in lower vertebrates as it is in mammals. Unfortunately, little is known about antigen-presentation in cartilaginous and bony fish. Indeed, such basic questions as where antigen-presentation takes place and which cells are primarily responsible for stimulating T cell proliferation are still unanswered. Because fish lack lymph nodes, the question of where antigen presentation takes place is of particular interest. We undertook the current studies to shed light on the nature of the cell type principally responsible for the initiation of adaptive responses in fish.

One of the obstacles to identifying APCs in fish is the dearth of specific antibodies available, although the reasons for this are not completely clear. It is thought that the heavily glycosylated surface of fish cells results in production of antibodies that bind to the glycosylated surface of all fish cells, rather than the specific antigen target [14]. Because of its high density on the cell surface and methods available to purify immunoglobulin (Ig), a monoclonal antibody to trout immunoglobulin M (IgM) was developed and characterized in 1983 [15]. This is the only well-characterized antibody that recognizes a defined antigen in trout.

Despite the absence of antibody reagents, several observations provide tantalizing evidence that cells homologous to mDCs exist in fish. These include the description in nurse shark of a network of MHC class II-positive cells in the T cell rich areas of the spleen [16]; a long-term trout splenic culture that yields non-adherent cells designated as DCs based on their morphology [17]; the identification of Birbeck-like granules in cells of the gill epithelium and lymphoid tissue of salmonids [18]; the description of a dendritic cell-like phagocytic cell line from Atlantic salmon [19]; and the staining of cells in the spleen and head kidney of rainbow trout and Atlantic salmon with a CD207/langerin (expressed on specialized skin mDCs called Langerhans cells) specific antibody [20]. While these observations make a case for the existence of DCs, the claim is tenuous without functional characterization of the relevant cell types, specifically with respect to antigen presentation. Two recent papers use the genetically tractable zebrafish (*Danio rerio*) model to address the existence of DCs. In the first, Lugo-Villarino et. al. described the existence of dendritic APCs in zebrafish based on morphology, staining characteristics, and functional assays involving the measurement of antigen specific recall responses [21]. The demonstration of an antigen specific recall response is a great step forward and clearly demonstrates the antigen processing and presenting capability of the cells described, but leaves the question open as to whether these cells are functionally equivalent to DCs, as it would be expected that all APCs would be capable of stimulating a recall response, but not a primary response. In further work from the same laboratory, reporter fish, with the expression of GFP controlled by MHCII regulatory elements and RFP expression under the control of the CD45 promoter, were developed in order to facilitate isolation of particular APC populations [22]. They found that GFP^hi^,RFP^hi^ cells were mature monocyte/macrophages and DCs, which were most abundant in the intestine and spleen. When exposed to bacteria, GFP^hi^,RFP^hi^ cells up-regulated inflammatory cytokines. These reporter fish will be invaluable for further characterization of mononuclear phagocytes in zebrafish.

In this paper, we functionally identify DCs in rainbow trout by demonstrating the existence of a population of cells that bear the essential hallmark of mammalian DCs: a superior ability to stimulate the MLR compared with other professional APCs. The identification of DCs in trout suggests that specialized APCs evolved in concert with the emergence of adaptive immunity in lower vertebrates.

## Materials and Methods

### Fish maintenance

Rainbow trout were obtained from Tunison Laboratory of Aquatic Science, Cortland, NY; New York State Department of Environmental Conservation Bath Fish Hatchery, Bath, NY; Carpenter's Brook Fish Hatchery, Elbridge, NY; and Fish Haven Farms, Candor, NY. All trout were maintained in flow-through tanks at an average temperature of 13°C. Fish were fed commercial trout chow once/day. This study was carried out in strict accordance with the recommendations in the Guide for the Care and Use of Laboratory Animals of the National Institutes of Health, and all efforts were made to minimize suffering. Animal maintenance and collection of tissues were conducted under protocol 1996-083 approved by the Institutional Animal Care and Use Committee of Cornell University.

### Hematopoietic Cell culture

200–2000 g rainbow trout were euthanized by overdose on MS-222 (Finquel, Redmond, WA). Fish were bled from the caudal vein prior to sterile removal of the spleen, head kidney, and anterior portion of trunk kidney, defined as a segment between the head kidney and Corpuscles of Stannius. Inclusion of kidney tissue posterior to the Corpuscles of Stannius resulted in excessive numbers of melanomacrophages in the cultures. Excised tissue was placed in supplemented media on ice. Supplemented media consists of L-15 media (Invitrogen, Carlsbad, CA) with 10% bovine growth serum (BGS, Hyclone, Logan, UT), gentamicin (50 µg/ml, Invitrogen, Carlsbad, CA), and fungizone (0.25 µg/ml, Invitrogen). Tissue was broken up with the stopper of a syringe and forced through a 70 µm cell strainer (BD, Franklin Lakes, NJ) resulting in a single cell suspension. Cells were counted and adjusted to a final concentration of approximately 6×10^6^ live cells/ml (as determined by trypan blue exclusion) in supplemented media and plated in 25 cm^2^ or 75 cm^2^ cell culture flasks with phenolic style caps (Corning, Corning, NY). Cells were cultured at room temperature for 7–30 days before harvest. In some cases, non-adherent cells could be harvested several times in that period replacing the removed medium with fresh supplemented media.

### Harvest and Enrichment of tDCs

Cell culture flasks were agitated to suspend non-adherent cells and the media was collected and pooled. The cell suspension was layered over Nycoprep/One-step monocytes (Accurate chemical, Westbury, NY) and spun as per manufacturer's instructions. The buffy coat, which was enriched for tDCs, was removed and washed twice in media prior to use.

### Serum collection

Euthanized trout were bled from the caudal vein under sterile conditions and the blood was allowed to clot at room temperature for 1–1.5 hours and then placed on ice. Clotted blood was then spun at 2000×g for 10 min at 4°C. Serum was collected and stored at −80°C. Serum samples were later thawed, pooled, diluted to 20% in L-15 media, and filter sterilized. The diluted, filtered serum was then heat-inactivated at 56°C for 20 min, aliquoted, and returned to −80°C until use.

### Phagocytosis Assay

Green fluorescent 1 µm latex beads (Sigma, St. Louis, MO) were opsonized by adding 5 µl beads per 100 µl L-15 medium supplemented with 10% trout serum and incubating for 1 hr at RT. Cells were suspended in supplemented media at a concentration of 1×10^7^ cells/ml and plated in 96 well flat bottom tissue culture plates (Corning, Corning, NY). An equal volume (100 µl) of opsonized beads was added to cells and plates were spun at 600×g for 2 minutes to bring the beads into contact with the cells. Following incubation for 2 hrs at RT, cells were washed 3× with PBS/0.1%BSA and re-suspended in 500 µl ice cold PBS/BSA until flow cytometry analysis. Initially, 0.4% trypan blue was added to tubes to quench the fluorescence of beads that had not been internalized, however, flow data collected from samples before and after addition of trypan blue were indistinguishable and the trypan blue addition was subsequently omitted. Cytospins also showed that beads not internalized were successfully removed in the wash steps.

### Flow cytometry

Cells were maintained at 4°C throughout staining protocol. Cells were blocked in 10% goat serum (Zymed, South San Francisco, CA) for 15 min, stained in primary antibody for 20 min, followed by secondary antibody for 20 min. Cells were washed 2× and re-suspended in 200 µL of ice cold PBS/0.5%BSA and placed on ice until flow cytometry was conducted. Data collection was performed on a FACSCalibur (BD, Franklin Lakes, NJ) using Cellquest software (BD). FloJo software was used for data analysis (Treestar, Ashland, OR). Primary antibodies used were: monoclonal antibody 1.14 [15] against rainbow trout IgM at 1∶500 (kind gift from Dr. Stephen Kaattari, Virginia Institute of Marine Sciences); anti-trout MHC class II β hyperimmune rabbit serum or pre-immune serum at 1∶4000; and anti-thrombocyte antibody S5H2 [23] at varying dilutions (neat, 1∶10, 1∶100, 1∶1000), and anti-thrombocyte antibody 28D7 [23] at 1∶200 (kind gifts from Dr. John Hansen, University of Washington/USGS-Western Fisheries Research Center). Secondary antibodies used were: goat anti-rabbit FITC (Invitrogen, Carlsbad, CA) at 1∶200; goat anti-mouse FITC (Invitrogen) at 1∶200; and goat anti-mouse AlexaFluor-633 (Invitrogen) at 1∶500.

### Microscopy and TEM

Images of cultures were taken on an Olympus CK2 inverted microscope with an Olympus SP-350 digital camera (Olympus, Center Valley, PA). Videos were generated from time-lapse images taken at 1-minute intervals over a two-hour time span. Cytospin micrographs were taken using a Zeiss Axioimager M1 microscope using Axiovision 4.6 software, and an Axiocam HR camera (Carl Zeiss, Germany).

Cytospins were stained with the Hema 3 stain set (Fisher Scientific).

Transmission electron microscopy was performed by the Cornell Integrated Microscopy Center. Double-strength glutaraldehyde (4%) was added to an equal amount of trout culture cells in suspension. After the primary fixation, the cells were washed three to six times (10 min each) in 0.1 M sodium cacodylate, 7.4 pH, and post-fixed in 1% osmium tetroxide. The material was washed as above in 0.1 M sodium cacodylate and dehydrated in a graded alcohol series. Following dehydration the material was infiltrated with plastic and then imbedded in blocks. Approximately 70 nm sections were taken with a Reichert OmU2 ultramicrotome. The sections were then contrasted using uranyl acetate and lead citrate. The grids were viewed in a Tecnai 12 Biotwin microscope; the images were acquired using a Gatan Multiscan camera, model 791.

### RNA preparation and RT-PCR

RNA was extracted from tissues in either Trizol (Invitrogen, Carlsbad, CA) or RNeasy buffers (Qiagen, Valencia, CA). Trizol extractions were performed as per the manufacturer's directions except for the inclusion of an additional phenol step (Ambion, Austin, TX). Following ethanol precipitation, RNA was dissolved in H_2_O and treated with RNase-free DNase (DNA-Free Turbo kit, Ambion) according to the manufacturer's protocol, then stored at −80°C. RNeasy extractions were performed according to the manufacturer's protocol. RNA eluted from columns was subjected to DNase treatment as described above. In all cases, RNA was quantitated by UV-spectrophotometry and subjected to electrophoresis on agarose gels to verify integrity. For RT-PCR 500 ng total RNA per 200 µL reaction was reverse transcribed using the High Capacity RNA-to-cDNA kit (Invitrogen) according to manufacturer's directions. 0.2 µL of Phusion high fidelity polymerase (NEB, Ipswich, MA) and 3 µL of cDNA (or no RT control RNA) template were used in 20 µL PCR reaction consisting of 30 s denaturing at 98°, followed by 35 cycles of 98° for 10 s, 50° or 63° for 15 s, 72° for 15 s, and a final extension at 72° for 5 minutes. PCR products were run on 2% agarose gels and were of the expected size. No PCR products were detected in controls that were not reverse transcribed. Primer sequences, product sizes, and annealing temperatures used in PCR are shown in Table S1.

### Real Time RT-PCR

Primers (Sigma Genosys, The Woodlands, TX) and probes (5′ 6-FAM, 3′ TAMRA labeled, Eurogentec, San Diego, CA) with the following sequences were designed with Primer Express software (Applied Biosystems, Foster City, CA): CD83 forward AGCAGAGGAACATGTCGATGC; CD83 Reverse GGCCAACCGATG-TTCAAAAT; CD83 probe AATTGCCTTCTACATTTGCCTGACCTTGATTT; MHCII forward AGCAGAGGAACATGTCGATGC; MHCII reverse GGCCAACCGATGTTCAAAAT; and MHCII probe AATTGCCTTCTACA-TTTGCCTGACCTTGATTT. Sequences used for primer and probe design were accessions: AY263797 (CD83), and U20947.1 (MHCII). For absolute quantitation, RNA transcripts were generated *in vitro* following standard protocols, diluted in yeast tRNA (6.25 ng/µl), and stored in single use aliquots at −80°C until use. One-step real time RT-PCR was done on an ABI 7500 Fast Real-Time PCR System (ABI, Foster City, CA) using the following conditions: 30 min 48°C for RT, 10 min 95°C for polymerase activation, followed by 40 cycles of 15 s at 95°C, then 1 min at 60°C. Reactions were performed in triplicate (for samples and duplicate for standards) in 96-well optical plates (ABI), using 17 µL master mix (one-step master mix [ABI] plus: 0.3 µM forward primer, 0.3 µM reverse primer, and 0.2 µM probe) and 8 µL of: RNA sample (50 ng total RNA), standard, or water in the case of no template controls. To control for genomic DNA contamination real time RT-PCR was done on each sample without the addition of reverse transcriptase. Product was not detected in the absence of template in control wells. Data analysis was done with ABI's built-in Sequence Detection System (v.1.4). The t test was used for statistical analysis.

### TLR-ligand treatment

Cells were stimulated for 24 hrs with a mixture of ssRNA (2 µg/mL), Imiquimod (10 µg/mL), Flagellin (from *S.typhimurium*, 100 ng/mL), and poly I:C (25 µg/mL) (all from Invivogen, San Diego, CA) in supplemented L-15 medium.

### Isolation of cells for mixed leukocyte reaction

IgM-positive (B cell stimulators) and IgM-negative (responders) splenocytes: Spleens were aseptically collected from euthanized trout and forced through a 70 µm cell strainer to form a single cell suspension. Cell suspensions were then fractionated on Accupaque medium according to the manufacturer's instructions (Accurate Chemical, Westbury, NY). After centrifugation, the buffy coat containing the lymphocyte fraction was removed and pooled. For MACS sorting into IgM-positive and IgM-negative fractions, this fraction was stained as for flow cytometry using (anti-IgM) mAb 1.14 [15] and FITC-labeled secondary antibody, then washed and incubated with MACS® anti-FITC beads (10 µL/10^7^ cells) for 15 min. Cells were re-suspended in 3 mL PBS/0.5%BSA and MACS® sorted using midi-MACS columns (Miltenyi Biotec, Germany). IgM-positive (B cell) and IgM-negative (responder) fractions were collected, washed, and counted for use in subsequent experiments.

Resident Peritoneal Macrophages: Peritoneal lavage fluid was collected, spun at 400×g for 10 min and the resulting cell pellet was re-suspended in 5–15 ml supplemented media. Cells were plated in a 6-well plate (Corning, Corning, NY) and incubated at room temperature from 2 hr to overnight depending on the experimental requirements. Non-adherent cells were washed off and the remaining adherent macrophages collected by incubating on ice in PBS/BSA without Ca^++^ or Mg^++^ for 10 min and harvesting with a cell scraper (Corning, Corning, NY).

### MLR

IgM-negative responder cells and stimulator cells (B cells, tDCs, and macrophages) were counted and re-suspended at a concentration of 1×10^6^cells/ml in PBS. Responders were labeled with 10 µM CFSE (Invitrogen, Carlsbad, CA) for 15 minutes, while stimulators were labeled with 10 µM Celltrace Far Red (Invitrogen) for 20 min. Five volumes of ice cold L-15 was then added to stop labeling and cells were incubated on ice for 5 min, followed by 2 washes in PBS. Cells were re-suspended at 2×10^6^cells/ml in L-15 medium containing gentamicin and fungizone (as above) and supplemented with 5% trout serum (MLR medium). Stimulators were then plated in 100 µl MLR medium at 2×10^5^ cells per well in 96-well round-bottom plates (Corning). A total of 2×10^5^ responders were then added to all wells resulting in a stimulator to responder ratio of 1∶1 (ratios of 1∶2, 1∶4, and 1∶8 were initially used). Negative control wells contained 4×10^5^ responders alone. MLR plates were incubated for 6 days (determined to be optimal in a time course experiment carried out to day 8). On day, 6 cells were re-suspended in PBS/BSA for flow cytometry. Data were collected and analyzed by excluding far red-positive cells (stimulators) in order to differentiate divided responders (CFSE-low) from CFSE-negative stimulators. Negative controls (responders alone) were used to draw a gate for dividing cells as any division seen in these wells was considered background (gate set at approximately 2.0% dividing cells).

### Transfer Experiments

Enriched tDCs were stained as above with CFSE for 20 min then washed 2× in L-15, followed by a final wash in PBS. A total of 2–4×10^7^ labeled cells was injected i.p. per fish in ∼250 µL PBS. Twenty-four hours after injection, fish were euthanized and blood, skin, gill, spleen, gut, head kidney, kidney, liver, and swim bladder tissue as well as peritoneal lavage fluid were collected in PBS/BSA/EDTA and placed on ice. Single cell suspensions were generated by passage through a 70 µm cell strainer (BD Falcon, Bedford, MA, USA). Certain tissues required additional treatment prior to passing through a cell strainer: spleen was treated with collagenase A (100 U/ml, Sigma, St. Louis, MO) at room temperature; swim bladder was rinsed in PBS, cut open, and cells were scraped off the interior surface with a scalpel and placed in media on ice. The remainder was cut up and then placed in PBS/0.5%BSA/Trypsin/EDTA/collagenase A (digestion solution) (Invitrogen; Serological Proteins Inc, Kankakee, IL; 0.5%, Invitrogen, Carlsbad, CA; and 100 U/ml, Sigma respectively). Similarly, gut was repeatedly rinsed out with PBS/BSA using a 10 mL serological pipette, cut open, and the surface cells were scraped off with a scalpel and placed on ice. The remaining tissue was cut up and placed into digestion solution. Skin (cut into small pieces) and gill (whole arches) were also placed in digestion solution. Incubations in digestion solution were on a shaker at room temperature for approximately 1–1.5 hours before generation of a single cell suspension. Suspensions from each tissue were strained though a 40 µm cell strainer (BD Falcon, Bedford, MA, USA) prior to flow cytometry. 1×10^6^ events (cells within live gate based on FWD-SSC scatter and PI exclusion) were collected per tissue. The un-migrated CFSE positive cells remaining in the peritoneal cavity were used to generate a gate, which was used to determine the number of CFSE positive cells in each tissue per one million events. The number of positive cells that had migrated was totaled for each fish and the percent of that total was determined for each tissue to arrive at a % migrated cells per tissue. A one-way ANOVA with Tukey post-test was used for statistical analysis.

### Spleen DC isolation and peripheral blood derived cultures

The protocol for isolation of DCs from spleen was carried out essentially as described for mammals [24]. Spleens were removed sterilely from euthanized trout and injected with collagenase A (100 U/ml, Sigma, St. Louis, MO) to facilitate dissociation and recovery of adherent cells. Single cell suspensions were spun over Nycoprep (Axis Shield, Oslo, Norway), a cell separation medium of similar density to bovine serum albumen,, according to the manufacturer's directions. The buffy coat was then removed and washed in media. Cells were counted, re-suspended, and plated in 6 well plates at a concentration of 1×10^6^ cells/ml in supplemented L-15 media. Cells were allowed to adhere for two hours, non-adherent cells were washed off, and new media was added. The cultures were incubated overnight, during which time the DCs lose their adherence and come up off the plates.

After experimentation the following protocol, adapted from mammalian methods [8], was used to generate peripheral blood cultures: Blood was drawn into heparin (10,000 Units/mL, Fisher Scientific, Pittsburgh, PA) from the caudal vein of multiple rainbow trout and pooled. The pooled whole blood was diluted four fold in PBS/heparin and allowed to settle for at least 20 minutes at room temperature. Leukocyte rich plasma was removed from settled red blood cells and spun over Nycoprep 1.077 (Axis Shield, Oslo, Norway) according to the manufacturer's directions. After centrifugation, the buffy coat was removed and plated in 25 cm^2^ flasks with phenolic caps at a density of approximately 5×10^6^ cells/ml of supplemented L-15 medium (20% FBS, 50 µg/mL gentamicin, and 0.25 µg/mL fungizone). Cells were allowed to adhere for two hours, at which point non-adherent cells were washed off and new media was added containing mammalian IL-4 (250 U/ml, human: Prospec, Israel; murine: Peprotech, Rocky Hill, NJ) and GM-CSF (1000 U/ml, human: Prospec,; murine: Peprotech). Cells were “fed” additional IL-4 and GM-CSF (same concentration as above) on days two, four, and six. Non-adherent cells removed at the 2 hr time point were transferred to a new flask for culture without addition of exogenous IL-4 or GM-CSF (or removal of non-adherent cells). These mixed cultures (sans cytokines) yielded much the same result as the adherent culture with cytokines. A more direct comparison of adherent cultures with or without exogenous cytokines was made. No discernable differences were seen (except perhaps small differences in the kinetics of the cultures), and addition of cytokines was subsequently abandoned. Cells were cultured at RT in the atmosphere.

## Results

### Hematopoietic cultures yield non-adherent cells with dendritic morphology

**Figure 1:**
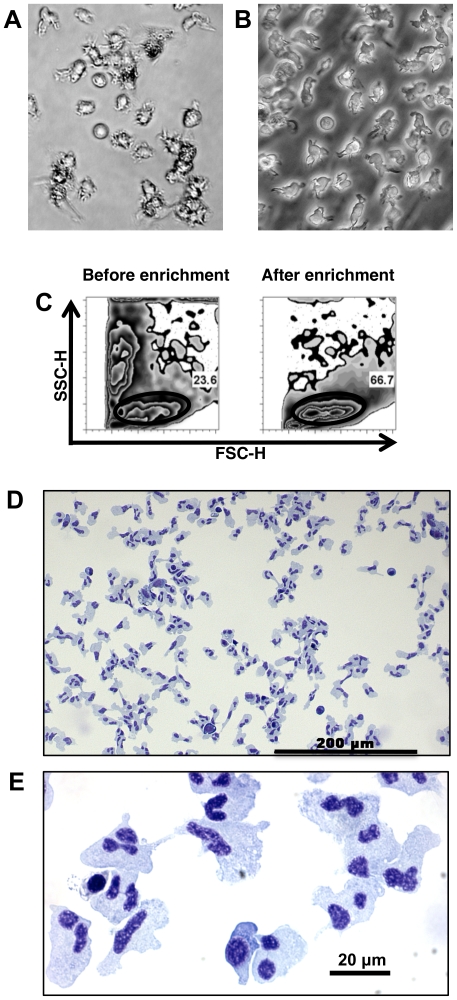
Culture and enrichment of tDCs. Phase bright (*A*) and phase contrast (*B*) images of hematopoietic cultures showing non-adherent cells with dendritic morphology. Flow cytometry forward/side scatter profile of cultured cells before and after enrichment (*C*). Gates indicate tDC population; typical purity after enrichment was 70–80%. (*D*) Low magnification of Giemsa-Wright stained cytospin of enriched cells. (*E*) High magnification of Giemsa-Wright stained cytospin of enriched cells.

In mice, DCs are commonly cultured from bone marrow in order to obtain the numbers needed to perform functional assays [7]. Because fish do not have bone marrow (hematopoiesis occurs in the kidney and spleen) we adapted mammalian protocols and began with primary cultures of head kidney, anterior trunk kidney and spleen of rainbow trout. Within twenty-four hours, many of the cells had adhered to the flask and formed clumps. Adherent cells were comprised primarily of melanomacrophages and macrophages. Small, round, non-adherent lymphocyte/thrombocyte cells were also present in variable numbers and persisted throughout. In addition to non-adherent lymphocytes and thrombocytes, larger, round, non-adherent monocyte-like cells were present in the culture that increased in number over time. By two weeks, these cells had assumed a branched morphology suggestive of the DCs of mammals (Fig. 1A–B) [1].

As is the case with mDCs, these branched cells had a low buoyant density and could be enriched by isopycnic separation on 1.068 g/mL separation medium [24]. Prior to separation, primary cultures contained mixtures of cells that could be readily distinguished by flow cytometry forward/side scatter profile (Fig. 1C). After enrichment cells became more uniform in size and complexity, with the major population having a similar forward/side scatter profile to that of monocytes, macrophages, and DCs in mammals (Fig. 1C) [25]. A forward/side scatter gate (Fig. 1C) on these cells indicates an average purity of 70–80% after enrichment. Cytospins of enriched cells show a homogenous population with irregular shape and lobular nuclei, similar to cells seen in mammalian DC cultures (Fig. 1D &E) [7], [26]. In particular, the presence of cells with multiple nuclear lobes joined by thin connections often in a clover leaf or flower petal arrangement were characteristic of the trout cultures. When compared to published micrographs of mDC cultures, the same nuclear morphology was prevalent [7], [26], [27].

### MHCII expression and mixed leukocyte reaction

**Figure 2:**
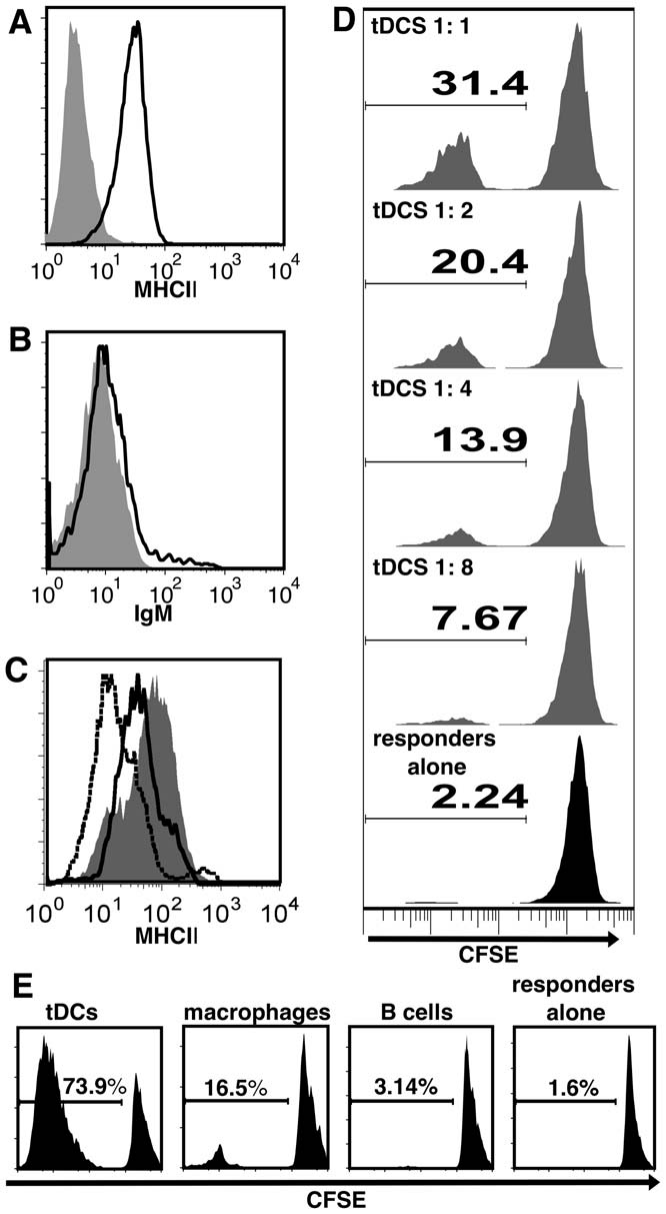
tDCs express MHCII and stimulate the MLR more effectively than B cells or macrophages. (*A*) tDCs are positive for surface MHCII by flow cytometry (black line histogram: MHCII hyper-immune serum, grey filled histogram: pre-immune serum control), but not IgM (*B,* black line histogram: anti-IgM, grey filled histogram: secondary antibody alone). (*C*) When compared to resting peritoneal macrophages (black dotted line histogram) and B cells (black solid line histogram), tDCs (solid grey histogram) express higher levels of MHCII on their surface. (*D*) tDCs were cultured with CFSE stained spleen responders in a primary allogeneic MLR at tDC to responder ratios from 1∶1 to 1∶8. Numbers represent percent dividing responders. (*E*) Allogeneic primary MLRs were conducted with tDCs, macrophages, or B cells as stimulators. Percent dividing cells was determined by flow cytometry analysis of CFSE dilution. Data are representative of 3 independent experiments, each using stimulators and responders from multiple individual fish.

Flow cytometry revealed that enriched cells expressed surface MHCII (Fig. 2A), but not immunoglobulin M (Fig. 2B). We therefore had a morphologically homogenous population of non-adherent cells that were not B cells (IgM-negative), but expressed surface MHCII, supporting our hypothesis that these cells could function as APCs. By way of comparison, resident peritoneal macrophages and splenic B cells were also stained for MHCII. Enriched cells expressed more MHCII on their surface than macrophages or B cells (Fig. 2C), supporting our hypothesis that these cells might function as DCs.

The defining feature of mDCs is their unrivaled efficacy in activating naive T cells. This was first demonstrated using the primary MLR, which continues to be the gold standard of APC function [2], [28]. In order to test the ability of enriched cells to present antigen we therefore performed a primary MLR. When enriched cells were mixed with allogeneic responders they could indeed stimulate division in a dose-dependent manner (Fig. 2D), although optimal ratios were higher than those reported for mDCs [2]. When the ability of enriched cells, B cells, and macrophages to stimulate the same responders was directly compared, the enriched cells consistently stimulated more proliferation in responders (Fig. 2E). Our data, therefore, demonstrate that the enriched cells are the functional equivalents of mDCs. Having thus functionally identified trout DCs (tDCs), we sought to more fully characterize these cells based on other known attributes of mDCs.

### Ultrastructure and motility of tDCs

**Figure 3:**
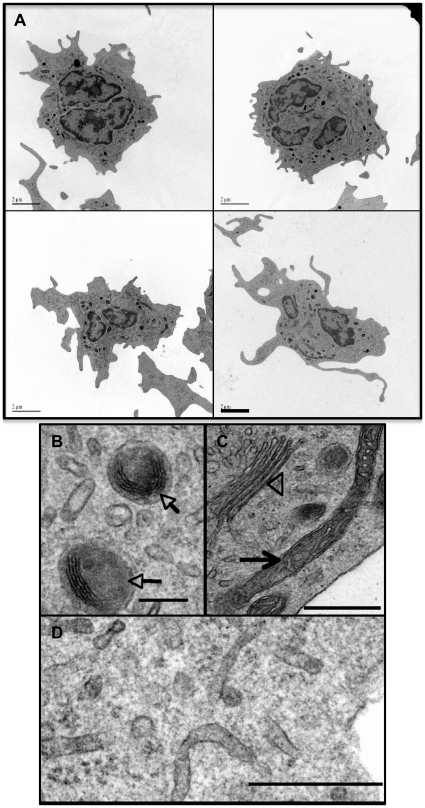
Ultrastructural features of tDCs. (*A*) Transmission electron micrographs of representative tDCs (scale bars = 2 µm). (*B*) High magnification of tDC cytoplasm detailing lamellar structures seen in some cells (arrows). (*C*) High magnification of tubular mitochondria (arrow), Golgi (arrow head) and smooth ER (*D*) characteristic of tDCs. Scale bar in *B* = 0.2 µm, *C* and *D* = 0.5 µm.

We performed transmission electron microscopy (TEM) to gain a more detailed view of these cells. Multi-lobular nuclei with condensed chromatin at the margins were typical, as were long cell processes that extended many microns from the periphery (Fig. 3A). tDCs were also rich in tubular ER and had characteristic vesicles containing electron dense material or in some cases, lamellar bodies (Fig. 3B–D). We did not observe any evidence of Birbeck granules. Consistent with mDCs, tDCs had irregular nuclei with a thick rim of heterochromatin and the presence of multivesicular bodies and many small smooth vesicles [1], [29], [30].

mDCs are highly motile and continually “taste” their environment by extending and contracting cytoplasmic veils and processes [1]. Consistent with this behavior, tDCs showed active motility, extending and retracting processes as they moved (Movie S1). In contrast, adherent macrophages demonstrated amoeboid-like movement that was uncharacteristic of tDCs (adherent cells in Movie S1).

### Phagocytosis

**Figure 4:**
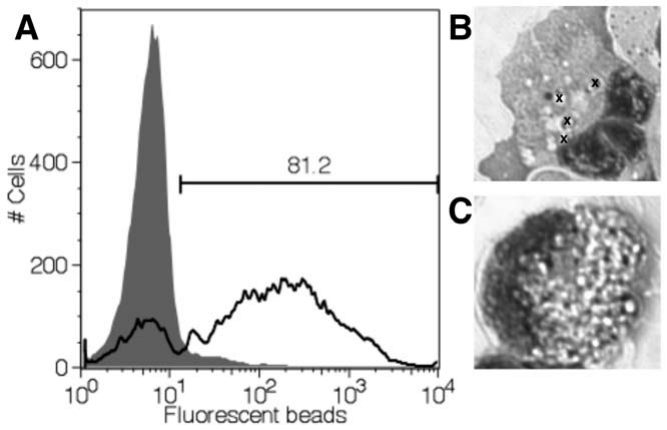
tDCs are phagocytic. (*A*) Fluorescence of tDCs incubated for 2 hours with 1 µM opsonized fluorescent latex beads (black line) compared to tDCs alone (grey filled histogram). Gate indicates percent phagocytic cells (81.2%). Representative data are shown (mean percent phagocytic tDCs for experiment was 68.6%, n = 5). Experiment was repeated 2 times. (*B*) Cytospin showing internalized beads (beads indicated by “x”) in a representative tDC, compared to a representative macrophage (*C*) under the same conditions (too many beads to mark).

In mammals, immature DCs are phagocytic but lose this ability upon activation with toll-like receptor (TLR)-ligands. tDCs were examined for their ability to take up 1 µm fluorescent beads. After two hours of incubation with opsonized beads, a majority of tDCs had phagocytosed beads, indicated by increased fluorescence (Fig. 4A) and an increase in side scatter (data not shown). tDCs phagocytosed limited numbers of beads per cell (Fig. 4B) unlike macrophages, which, under the same conditions filled their cytoplasm to capacity with beads (Fig. 4C).

### Migration in vivo

**Figure 5:**
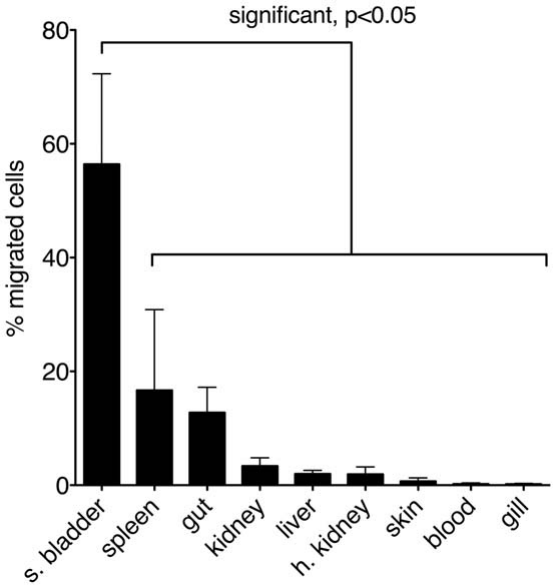
tDCs migrate in vivo. tDCs were stained with CFSE and injected intraperitoneally into recipient fish. Twenty-four hours later, the number of CFSE positive, live events in one million collected events per tissue was determined by flow cytometry. The number of CFSE positive events in all tissues was totaled and events per tissue were divided by this total to arrive at percent of migrating cells in each tissue. Data are from four independent experiments. Error bars indicate SEM.

mDCs are located in the periphery, most notably in the skin and mucosal surfaces, where they encounter pathogens, become activated and consequently migrate to secondary lymphoid tissue (spleen and lymph nodes), where they present antigen to T cells. Sites of antigen presentation in fish have yet to be characterized, partly because fish lack lymph nodes. In the absence of lymph nodes, the spleen (because of its similar organization to mammals) and kidney are considered likely sites of T cell–APC interactions. To determine if tDCs migrate *in vivo*, either to proposed sites of antigen presentation or to peripheral sites, we transferred CFSE-labeled tDCs to allogeneic fish. Twenty-four hours after intraperitoneal injection, the number of viable CFSE-positive cells in various tissues of recipient fish was ascertained by flow cytometry. Migration of injected tDCs to tissues was observed. Surprisingly, a significantly higher percentage of CFSE-positive cells were recovered from the swim bladder than any other tissue examined (Fig. 5). The swim bladder is evolutionarily homologous to the lung, and in rainbow trout has an opening into the esophagus that allows exchange with the external environment. It is not clear whether this mucosal tissue is a site of immune interaction with the external environment as is the case with the mammalian lung. Although not statistically significant, higher percentages of cells were recovered from spleen and gut; two sites that are rich in DCs in mammals. While we cannot exclude the possibility that some tDCs were phagocytosed by other cells and these phagocytic cells were detected as CFSE+, we think it is unlikely that this is true of all CFSE+ cells recovered. We therefore hypothesize that tDCs preferentially migrate *in vivo* to mucosal (gut and swim bladder) and lymphoid (spleen) tissue. The development of a specific antibody would make it possible to test this in a more rigorous manner.

### Surface marker and mRNA expression

Few specific trout antibodies are available to characterize the identity of tDCs beyond IgM and MHCII, however, anti-thrombocyte antibodies have been characterized and thrombocytes have been reported to express MHC class II transcripts [23] in trout, which could indicate an APC function for these cells. tDCs failed to stain with these antibodies, indicating that tDCs are not thrombocytes, but a distinct subset of cells (data not shown).

**Figure 6:**
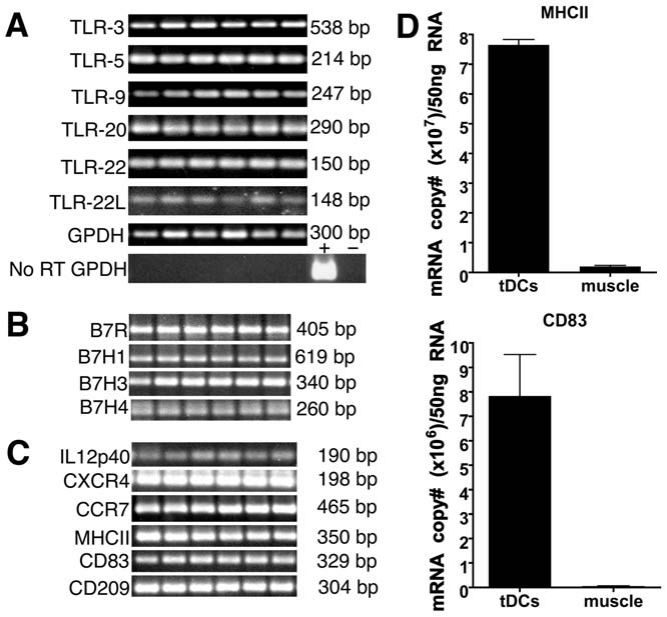
DC marker expression. RT-PCR was performed for trout homologs of mammalian DC markers of interest. (*A*) tDCs express mRNA for TLR-3, -5,-9,-20,-22, and-22L. (*B*) tDCs express mRNA for the B7 costimulatory molecules B7R, B7H1, B7H3, and B7H4. (*C*) tDCs express mRNA for molecules involved in DC function including: IL12p40, CXCR4, CCR7, MHCII, CD83, and CD209. Results for tDCs from six individual fish are shown. PCRs shown were performed in parallel. No PCR products were detected in no RT controls or no template controls (GPDH no RT controls shown, other data not shown). (*D*) Quantitative real-time RT-PCR analysis of CD83 and MHCII mRNA expression in tDCs compared to muscle (error bars indicate SD, *p<0.05).

In the absence of additional specific antibodies, RNA was isolated for RT-PCR analysis with a panel of primers for markers of interest. The caveat to RT-PCR analysis is that it cannot be determined if a majority of cells or a small fraction are responsible for the detected mRNA, however, particularly in the absence of antibodies it is useful to know if mRNAs of interest could be detected. As shown in Figure 6A, tDCs expressed transcripts for TLRs including: TLR-3, TLR-5, TLR-9, TLR-20, TLR-22, and TLR-22L suggesting that, like mDCs, tDCs can sense TLR-ligands.

The specialized APC function of DCs is largely a result of their ability to express high levels of costimulatory molecules. Until very recently, rainbow trout costimulatory molecules had eluded identification, however, a B7-like molecule (B7R) has now been recognized in trout as well as homologs to B7H1, B7H3, and B7H4 [31], [32]. tDCs expressed all four costimulatory molecules, supporting their role as specialized APCs (Fig. 6B). tDCs also expressed transcripts for other markers involved in DC function including: CXCR4 and CCR7, chemokine receptors expressed by mDCs [33], [34]; CD209/DC-SIGN, a C-type lectin expressed on mDCs [35]; CD83, an Ig family member expressed on mDCs [36]; and IL-12p40, a component of cytokine produced by mDCs [37] (fig. 6C). We designed real time RT-PCR primers for two molecules of particular interest, CD83, and MHCII. When examined by real time quantitative RT-PCR, tDCs expressed abundant levels of CD83 and MHCII mRNA transcripts compared with muscle, a tissue expected to be relatively free of antigen-presenting cells (Fig. 6D).

### Activation by toll-like receptor-ligands

**Figure 7:**
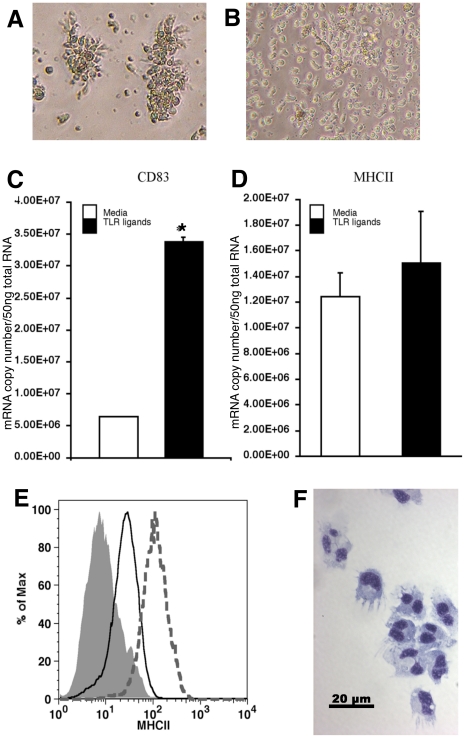
Activation of tDCs by TLR-ligands. Phase contrast images show aggregation of tDCs typical of activated leukocytes after 24 hrs incubation with TLR-ligands (*A*), while tDCs in medium alone remained randomly distributed (*B*). tDC CD83 mRNA copy numbers, measured by real-time RT-PCR, increase significantly (*p<0.05) compared to media controls at 24 hrs of exposure to TLR-ligands (*C*), while MHCII mRNA copy numbers are not significantly changed (*D*). However, after 4 day incubation with TLR-ligands, tDCs upregulate surface expression of MHCII (*E*), (grey dotted histogram: TLR-ligand treated tDCs, black line histogram: media control tDCS, and grey filled histogram: pre-immune serum control) and by cytospin take on a morphology strikingly similar to mammalian DCs (*F*).

The unique activation program of mDCs is triggered by exposure to TLR-ligands and other PAMPs. Our data indicate that tDCs express TLR-3, -5, -9, -20, -22, and -22L, which suggests that these cells can respond to TLR-agonists. Trout TLR-5 is reported to recognize flagellin, but ligand specificity of other trout TLRs has yet to be elucidated [38]. While TLR signaling is well conserved from fish to mammals in general, it appears that specifically LPS signaling is not well conserved [39]. Many fish species do not have TLR-4 orthologs or the signaling molecules involved in LPS recognition and signaling [40], [41]. Fish are not susceptible to endotoxic shock, and in most studies 1000-fold higher concentrations of LPS than those used in mammals are required to elicit a response from fish cells [32], [42], [43], [44], [45]. Therefore, we treated tDCs with a mixture of four TLR-ligands (imiquimod, Poly I:C, ssRNA, and flagellin) to generate a strong response akin to those described for mammalian responses to LPS. After twenty-four hours, tDCs showed marked aggregation (Fig. 7A), while cells in media alone remained randomly distributed (Fig. 7B). Aggregation is characteristic of activated leukocytes and has been described in mammals and trout alike [46], [47]. At the molecular level, significant increases in CD83 mRNA transcripts were observed in tDCs treated with TLR-ligands compared with media controls (Fig. 7C). This, in addition to aggregation, suggested tDCs are activated by TLR-ligands. Interestingly, no increases in the expression of MHC class II transcripts were observed at the twenty-four hour time point (Fig. 7D). Surface MHC class II expression was also unchanged (data not shown).

To examine MHCII expression in response to TLR-agonists further, we performed time course studies and found that tDC MHC class II surface expression increased after 4 days of TLR-ligand treatment (Fig. 7E). When cytospin preparations were made of these cells, they bore a striking resemblance to mDCs [7], [26] (Fig. 7F). TLR activated tDCs were large round cells with kidney shaped nuclei and trailing dendrites. The increases in surface MHCII and CD83 mRNA, along with changes in morphology, seen in tDCs treated with TLR-ligands is consistent with TLR activation of mDCs, but on a slower time scale [48], [49], [50].

### Isolation of spleen tDCs and culture from peripheral blood

**Figure 8:**
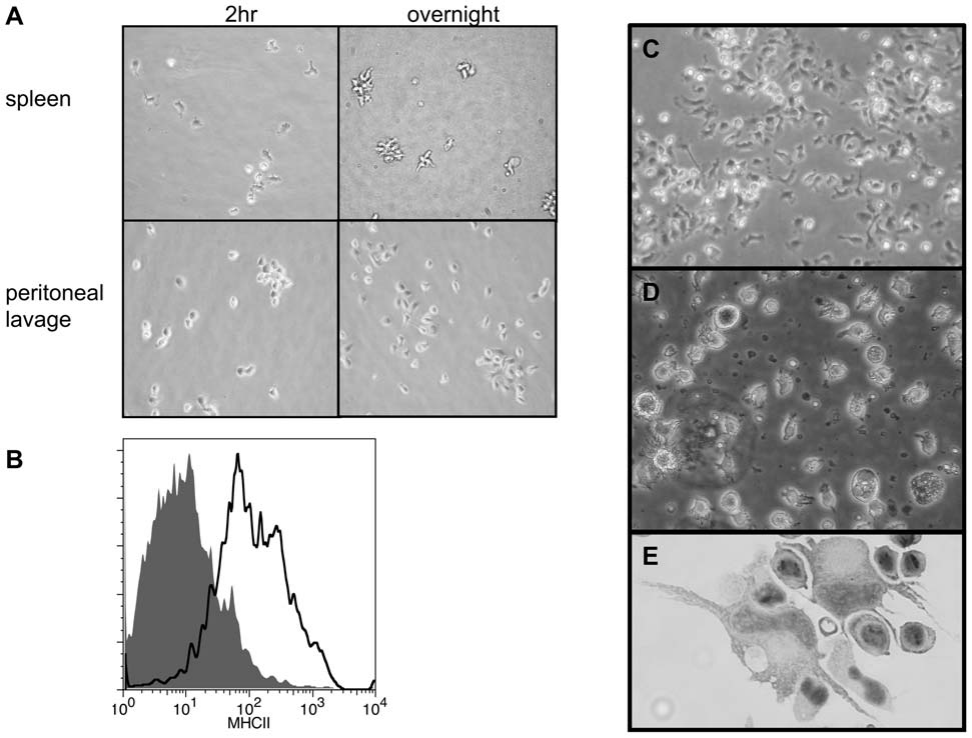
Isolation of tDC-like cells from the spleen and culture from peripheral blood mononuclear cells. (*A*) Phase contrast images of tDC-like cells isolated from spleen using a mammalian protocol. Isolated cells are initially adherent (2 hr) but become non-adherent overnight, unlike macrophages from peritoneal lavage that remain adherent. (*B*) Non-adherent cells isolated from spleen express surface MHCII (grey filled histogram: pre-immune serum, black line histogram: anti-MHCII hyper-immune serum). (*C*) Phase contract image of adherent cells from mononuclear fraction of peripheral blood after 2 hr incubation. (*D*) Phase contrast of typical culture of adherent peripheral blood cells (20×, day 18). (*E*) Cytospin of peripheral blood cultures reveal the presence of large cells with long veils and kidney shaped nuclei (40×, day 7).

To address the concern that tDCs could represent an artifact of culturing we used the mammalian protocol for isolation of DCs directly from the spleen to demonstrate the presence of these cells *in vivo*. We initiated our inquiries with primary cultures because of the rarity of DCs *in vivo*, however, mDCs can be directly isolated from spleen and lymph nodes in small numbers. The initial isolation of DCs in mammals was accomplished by collagenase treatment of spleen and plating of the low buoyant density fraction of cells [24]. DCs were initially adherent, with a stellate or branched morphology, but became non-adherent overnight [23]. Using these same techniques on trout spleen resulted in the same phenomena (Fig. 8A). The spleen cells had a distinct branched morphology while adherent, and overnight became floating cells with many processes that formed clumps. Peritoneal macrophages, on the other hand, retained the same rounded, adherent characteristics after overnight culture. Cells isolated from spleen were morphologically similar to tDCs, and when analyzed by flow cytometry, also expressed surface MHCII (Fig. 8B). Adequate numbers of cells to perform an MLR could not be isolated, but the acquisition of larger fish should allow for this. However, based on the behavior and morphology of these cells (which in mammals are unique to DCs), along with their MHCII expression, it is likely that these cells are tDCs.

In addition to bone marrow cultures, mDCs can be derived from peripheral blood mononuclear cells. The distinct advantage to this approach is that it does not require euthanasia, but the amount of peripheral blood that can be drawn is obviously limited. As described above (Materials and Methods), a protocol modified from the mammalian literature yielded non-adherent cells with DC morphology (Fig. 8D,E). Adherent peripheral blood mononuclear cells after two-hour incubation were very similar in appearance to adherent cells from spleens (Fig. 8A & C). After approximately one week, significant numbers of non-adherent, spiky cells are present in the culture (Fig. 8D). Cytospin preparations of these cultures showed these cultures contained cells with a striking resemblance to mDCs, having long trailing processes and large kidney shaped nuclei (Fig. 8E).

## Discussion

Under the conditions described here, single-cell suspensions from head kidney, anterior portion of trunk kidney, and spleen of rainbow trout give rise to populations of non-adherent cells with low buoyant density that are highly motile in culture. As with dendritic cells in mammals, when mixed with allogeneic spleen cells, tDCs induce potent proliferative responses compared to macrophages and B cells. As this is the functional definition of DCs in mammals, we conclude that tDCs serve a specialized APC function in fish analogous to the role of DCs in mammals. To our knowledge this is the first functional demonstration of DCs in fish. Further analyses suggest tDCs share other characteristics with mDCs as well. tDCs extend slender cytoplasmic dendrites, express surface MHCII, express TLR, costimulatory molecule, CD83, CD209, CXCR-4, CCR-7, IL-12p40, and MHC class II mRNAs, are phagocytic, and are activated by TLR-ligands. The kinetics of the activation of tDCs by TLR-ligands shows delayed kinetics when compared to mammals. However, several measures of immune responses in fish suggest that *in vivo* immune responses occur more slowly than in mammals [51], [52], [53].

The rationale behind the approach taken here is that DCs could be generated from fish tissues using the same methods employed for the production of bone-marrow derived DCs in mammals. Typically, this involves the addition of growth factors to bone marrow cells. Initially, we added recombinant mammalian GM-CSF to trout cell cultures but found that we could generate tDCs even in its absence. This is similar to long-term stromal cell cultures from mouse spleen that support the production of immature DCs without the requirement for exogenous sources of GM-CSF [54]. We therefore presume tDCs emerge from progenitor cells under the influence of endogenous growth factors present in cultures. As noted in the text, tDCs appear to develop from non-adherent monocytic cells in culture. However, when these cells were removed and the media replenished, tDCs continued to emerge for up to four months indicating that they may arise from adherent precursors in addition to non-adherent monocytic progenitors.

Although the studies reported here describe cultures from head kidney, anterior portion of trunk kidney, and spleen tissues combined, tDCs could also be generated from each of those tissues individually, as well as from the posterior portion of the trunk kidney. Larger numbers of melanomacrophages (along with unidentified debris) appeared in cultures containing posterior portions of the trunk kidney, and therefore these areas of the kidney were avoided. Interestingly, similar to mDCs, cells with the same morphology as tDCs could also be generated from peripheral blood mononuclear cells of rainbow trout or isolated from spleen using methods from the mammalian literature. These cells expressed MHCII, but functional studies to determine whether these cells are also capable of stimulating the MLR have yet to be performed.

tDCs possess distinctive multi-lobed nuclei that superficially resemble the nuclei of polymorphonuclear cells (PMNs). PMN nuclear lobes however, are circular in arrangement, while tDCs most often display a clover leaf-type arrangement with multiple lobes linked at a central point by thin extensions of nuclear material. Early publications describing mammalian DC cultures show cells with remarkably similar nuclear morphology to tDC cultures that do not stain with granulocyte antibodies, suggesting that these cells are a maturation stage of DCs in culture [7], [26], [27]. Additionally, tDCs are not highly phagocytic, as would be expected of PMNs. tDCs do phagocytose beads, but the cells do not fill to capacity as is typically observed for PMNs and macrophages. The scarcity of granules in tDCs also supports our conclusion that these cells are not PMNs, and respresent a distinct subset of cells.

While only a limited number of specific antibodies are available for delineating cell types in fish, our data indicate that tDCs fail to bind either IgM or thrombocyte-specific antibodies. In contrast, rabbit antisera against rainbow trout MHC class II bound uniformly to tDCs. In addition to the surface expression of MHC class II, we show that CD83 and MHC class II genes are expressed in these cells using real-time RT-PCR. Surface protein expression of CD83, a specific marker for mature DCs in mammals, has recently received a great deal of scrutiny as its function is unraveled. While the full story has not been elucidated it does appear that its expression is correlated with MHC class II surface expression [55]. The pattern of CD83 mRNA tissue expression is reported to correlate with that of MHC class II mRNA in rainbow trout, suggesting that, like mammals, CD83 and MHC class II expression may be correlated [56]. CD83 gene expression has been reported in fish leukocytes including trout macrophages (where it is reportedly upregulated in response to LPS) [57], [58], the RTS11 macrophage-like cell line [59], a melanin-producing leukocyte cell line [60], and the Atlantic salmon phagocytic TO cell line [19], as well as endothelial cells [61]. CD83 mRNA expression is reported in a range of mammalian cells as well, including: T cells [62], B cells [63], neutrophils [64], monocytes, and macrophages, but stable protein surface expression is seen only in DCs [65]. It is possible that CD83 mRNA expression is not a specific marker for DCs in fish; however, it should be noted that mRNA levels do not necessarily reflect surface expression of translated protein, and CD83 could undergo post-translational regulation in fish as it does in mammals [66]. Despite mRNA expression in cells that are not DCs in fish, surface expression of CD83 protein may still prove to be a specific marker for DCs in teleosts. Generation of a CD83 specific antibody to test this has yet to be achieved. It should also be noted that along with expression of CD83 mRNA, apparent expression of transcripts for other DC markers (TLR, co-stimulatory molecule, CD83, CD209, CXCR-4, CCR-7, and IL-12p40) in tDCs based on PCR is still somewhat tentative given the nature of the samples and the lack of data regarding the expression of these markers in other fish cell types.

Irrespective of the role of CD83 in antigen-presentation, we found that CD83 mRNA transcripts, along with surface levels of MHC class II were upregulated in tDCs in response to addition of TLR-ligands to culture media. Upregulation of the expression of surface MHC class II and CD83 mRNA transcripts are consistent with the activation pattern seen in TLR-ligand treated mDCs and has not been shown in fish cells before. To examine this further, we investigated whether the phagocytic capacity of tDCs was also altered in response to PAMPs. In particular, we were curious as to whether tDCs would become less phagocytic, as mDCs do, following activation via toll-like receptor signaling. Inconclusively, we found that phagocytosis in tDCs after TLR-ligand treatment increased in some experiments and decreased in others (data not shown). We hypothesize that differences in the maturation state of tDCs from culture to culture could account for the inconsistent response. Relatively immature cells could become more phagocytic with the addition of TLR ligands, taking longer to reach a mature state where they would lose their phagocytic capacity. On the other hand, cells that are relatively mature may be phagocytic and become less so with the addition of TLR-ligands. The maturation state of any given culture could depend on the relative levels of endogenous cytokines present, which in turn may vary from culture to culture. Additionally, it is important to bear in mind that trout used in these studies were from out-bred populations and were not housed in specific pathogen free conditions. In fact, spleens of euthanized fish were frequently enlarged, suggesting an on-going immune response. These factors are likely to contribute significant variation in the maturation status of leukocytes from individual fish. As with mDCs, the relative maturation state of tDCs could potentially be normalized by the addition of exogenous cytokines to cell cultures, or by treatment with TLR-ligands such as CpG [67]. In effect, our treatment of tDCs with TLR-ligands for four days resulting in surface MHCII upregulation in the ensuing population of cells suggests that tDCs can be matured by TLR-stimulation. Further studies on four-day stimulated tDCs are planned to characterize these cells, including assessment of changes in phagocytic capacity, as they may represent more uniformly activated tDCs.

MLRs have previously been demonstrated in several species of fish including rainbow trout [12], [68], [69], [70]. The seminal studies on APC function in fish were conducted in channel catfish. These studies established a role for “accessory cells” or APCs in fish by determining that Ig-negative lymphocyte (presumably T cell) responses to LPS or Con A required the presence of monocytes/macrophages, defined as peripheral blood leukocytes that adhered to baby hamster kidney cell microexudate-coated surfaces (presumed to be fibronectin) or Sephadex G-10 [71]. It was also observed that monocytes/macrophages, B cells, and to a lesser extent, Ig-negative lymphocytes, could stimulate the MLR, whereas Ig-negative lymphocytes were the only cells capable of acting as responders [72]. The number of monocytes/macrophages that could be isolated in these studies was limited, preventing a direct comparison with B cells, although monocytes/macrophages appeared to be better stimulators. The monocyte/macrophage fraction of peripheral blood leukocytes used in these studies may be or contain the channel catfish-equivalent of rainbow trout DCs. Studies to determine the relevance of tDC culture methods to other species of fish, such as catfish, are an important next step.

When compared, fish and mammalian immune systems have notable similarities although anatomically they clearly differ. Fish do not have bone marrow. The head kidney, and in some species (such as rainbow trout), both the head kidney and spleen are the bone marrow equivalents. Additionally, fish do not have lymph nodes, the sites of antigen presentation and germinal center formation in mammals. It is thought that antigen presentation occurs in the spleen and possibly head kidney in fish, but this has yet to be demonstrated. Formation of germinal centers has not been observed in fish. The splenic architecture of fish shows organized B cell and T cell areas, similar to mammals, suggesting that in the absence of lymph nodes, the spleen functions as an important secondary lymphoid tissue, as it does in mammals. Immune responses in mice that lack lymph nodes and have disrupted splenic architecture are surprisingly robust, although delayed [73]. Thus the lack of lymph nodes in fish may not represent a significant difference between fish and mammals in the way immune responses are mounted. In fact, the delayed immune responses seen in mice lacking functional lymph nodes invites conjecture that perhaps the comparatively slower immune responses in fish may also be partly attributable to their lack of lymph nodes.

This paper is an important advance in our understanding of how adaptive responses are initiated in fish, which is essential for rational vaccine design for aquaculture species. In mammals, direct targeting of antigen peptides to DCs has proven to elicit T cell responses that are superior to traditional antigen and adjuvant vaccines [74], [75]. Such approaches can be applied to fish vaccines, but rely on the identification of this cell type, as demonstrated here for rainbow trout.

Evolutionarily distant species have not traditionally been used as basic immunological models, however, information central to our current understanding of mammalian immunology has resulted from the study of such species. For example: the Toll gene was discovered in the invertebrate *Drosophila* in 1985, and was subsequently shown to have a dual role in development and the innate immune response [76], [77]. Toll-like receptors were consequently identified in humans in 1997 and have since been demonstrated to be one of the main modes of sensing pathogens by the mammalian immune system [78]. This important discovery, crucial to our current understanding of immunology, was based on work done in an invertebrate. Clearly study of our ancient vertebrate ancestors is relevant to our understanding of mammalian immunology. As with most things, it behooves us to start at the beginning.

## Supporting Information

Movie S1 **Motility of tDCs.** Time-lapse video shows refractile trout DCs moving actively from right to left across the field extending slender processes as they move. The more flattened macrophages near the center of the field show amoeboid like movement with broad lamellipodia at their leading edges.
(MPG)

Table S1 **Primers List.** Sequences and annealing temperatures of primers used for PCR amplification of immune-related and house-keeping (GPDH) genes from tDC cultures are listed, along with the expected sizes of amplicons for relevant primer pairs. Sources of primer sequences not listed in the references are indicated at the bottom of the table.
(DOC)

## References

[1] Steinman RM, Cohn ZA (1973). Identification of a novel cell type in peripheral organs of mice: I. Morphology, quantitation, tissue distribution.. J Exp Med.

[2] Steinman RM, Witmer MD (1978). Lymphoid dendritic cells are potent stimulators of the primary mixed leukocyte reaction in mice.. Proc Natl Acad Sci U S A.

[3] Muzio M, Polentarutti N, Bosisio D, Prahladan MK, Mantovani A (2000). Toll-like receptors: a growing family of immune receptors that are differentially expressed and regulated by different leukocytes.. J Leukoc Biol.

[4] Pierre P, Turley SJ, Gatti E, Hull M, Meltzer J (1997). Developmental regulation of MHC class II transport in mouse dendritic cells.. Nature.

[5] Guermonprez P, Valladeau J, Zitvogel L, Théry C, Amigorena S (2002). Antigen presentation and T cell stimulation by dendritic cells.. Annu Rev Immunol.

[6] Banchereau J, Briere F, Caux C, Davoust J, Lebecque S (2000). Immunobiology of dendritic cells.. Annu Rev Immunol.

[7] Inaba K, Inaba M, Romani N, Aya H, Deguchi M (1992). Generation of large numbers of dendritic cells from mouse bone marrow cultures supplemented with granulocyte/macrophage colony-stimulating factor.. J Exp Med.

[8] Sallusto F, Lanzavecchia A (1994). Efficient presentation of soluble antigen by cultured human dendritic cells is maintained by granulocyte/macrophage colony-stimulating factor plus interleukin 4 and downregulated by tumor necrosis factor alpha.. J Exp Med.

[9] Vallejo AN, Miller NW, Warr GW, Gentry GA, Clem LW (1993). Phylogeny of immune recognition: fine specificity of fish immune repertoires to cytochrome C.. Dev Comp Immunol.

[10] Vallejo AN, Miller NW, Clem LW (1991). Phylogeny of immune recognition: role of alloantigens in antigen presentation in channel catfish immune responses.. Immunology.

[11] Vallejo AN, Miller NW, Clem LW (1991). Phylogeny of immune recognition: processing and presentation of structurally defined proteins in channel catfish immune responses.. Dev Immunol.

[12] Vallejo AN, Miller NW, Jorgensen T, Clem LW (1990). Phylogeny of immune recognition: antigen processing/presentation in channel catfish immune responses to hemocyanins.. Cell Immunol.

[13] Secombes C (2008). Will advances in fish immunology change vaccination strategies?. Fish Shellfish Immunol.

[14] Miller N, Wilson M, Bengtén E, Stuge T, Warr G (1998). Functional and molecular characterization of teleost leukocytes.. Immunol Rev.

[15] DeLuca D, Wilson M, Warr GW (1983). Lymphocyte heterogeneity in the trout, Salmo gairdneri, defined with monoclonal antibodies to IgM.. Eur J Immunol.

[16] Rumfelt LL, McKinney EC, Taylor E, Flajnik MF (2002). The development of primary and secondary lymphoid tissues in the nurse shark Ginglymostoma cirratum: B-cell zones precede dendritic cell immigration and T-cell zone formation during ontogeny of the spleen.. Scand J Immunol.

[17] Ganassin RC, Bols N (1996). Development of long-term rainbow trout spleen cultures that are haemopoietic and produce dendritic cells.. Fish Shellfish Immunol.

[18] Lovy J, Wright GM, Speare DJ (2008). Comparative cellular morphology suggesting the existence of resident dendritic cells within immune organs of salmonids.. Anat Rec (Hoboken).

[19] Pettersen EF, Ingerslev HC, Stavang V, Egenberg M, Wergeland HI (2008). A highly phagocytic cell line TO from Atlantic salmon is CD83 positive and M-CSFR negative, indicating a dendritic-like cell type.. Fish Shellfish Immunol.

[20] Lovy J, Savidant GP, Speare DJ, Wright GM (2009). Langerin/CD207 positive dendritic-like cells in the haemopoietic tissues of salmonids.. Fish Shellfish Immunol.

[21] Lugo-Villarino G, Balla KM, Stachura DL, Banuelos K, Werneck MB (2010). Identification of dendritic antigen-presenting cells in the zebrafish.. Proc Natl Acad Sci U S A.

[22] Wittamer V, Bertrand JY, Gutschow PW, Traver D (2011). Characterization of the mononuclear phagocyte system in zebrafish.. Blood.

[23] Kollner B, Fischer U, Rombout JH, Taverne-Thiele JJ, Hansen JD (2004). Potential involvement of rainbow trout thrombocytes in immune functions: a study using a panel of monoclonal antibodies and RT-PCR.. Dev Comp Immunol.

[24] Steinman RM, Kaplan G, Witmer MD, Cohn ZA (1979). Identification of a novel cell type in peripheral lymphoid organs of mice. V. Purification of spleen dendritic cells, new surface markers, and maintenance in vitro.. J Exp Med.

[25] Crowley M, Inaba K, Witmer-Pack M, Steinman RM (1989). The cell surface of mouse dendritic cells: FACS analyses of dendritic cells from different tissues including thymus.. Cellular Immunology.

[26] Inaba K, Steinman RM, Pack MW, Aya H, Inaba M (1992). Identification of proliferating dendritic cell precursors in mouse blood.. J Exp Med.

[27] Lu L, Hsieh M, Oriss TB, Morel PA, Starzl TE (1995). Generation of DC from mouse spleen cell cultures in response to GM-CSF: immunophenotypic and functional analyses.. Immunology.

[28] Anandasabapathy N, Victora GD, Meredith M, Feder R, Dong B (2011). Flt3L controls the development of radiosensitive dendritic cells in the meninges and choroid plexus of the steady-state mouse brain.. J Exp Med.

[29] Van Voorhis WC, Hair LS, Steinman RM, Kaplan G (1982). Human dendritic cells. Enrichment and characterization from peripheral blood.. J Exp Med.

[30] Lu L, Woo J, Rao AS, Li Y, Watkins SC (1994). Propagation of dendritic cell progenitors from normal mouse liver using granulocyte/macrophage colony-stimulating factor and their maturational development in the presence of type-1 collagen.. J Exp Med.

[31] Hansen JD, Du Pasquier L, Lefranc MP, Lopez V, Benmansour A (2009). The B7 family of immunoregulatory receptors: a comparative and evolutionary perspective.. Mol Immunol.

[32] Zhang YA, Hikima J, Li J, LaPatra SE, Luo YP (2009). Conservation of structural and functional features in a primordial CD80/86 molecule from rainbow trout (Oncorhynchus mykiss), a primitive teleost fish.. J Immunol.

[33] Dieu M-C, Vanbervliet B, Vicari A, Bridon J-M, Oldham E (1998). Selective Recruitment of Immature and Mature Dendritic Cells by Distinct Chemokines Expressed in Different Anatomic Sites.. J Exp Med.

[34] Federica S, Patrick S, Pius L, Christoph S, Danielle L (1998). Rapid and coordinated switch in chemokine receptor expression during dendritic cell maturation.. Eur J of Immunol.

[35] Geijtenbeek TBH, Torensma R, van Vliet SJ, van Duijnhoven GCF, Adema GJ (2000). Identification of DC-SIGN, a novel dendritic cell specific ICAM-3 receptor that supports primary immune responses.. Cell.

[36] Zhou LJ, Tedder TF (1995). Human blood dendritic cells selectively express CD83, a member of the immunoglobulin superfamily.. J Immunol.

[37] Heufler C, Koch F, Stanzl U, Topar G, Wysocka M (1996). Interleukin-12 is produced by dendritic cells and mediates T helper 1 development as well as interferon-gamma production by T helper 1 cells.. Eur J Immunol.

[38] Tsujita T, Tsukada H, Nakao M, Oshiumi H, Matsumoto M (2004). Sensing bacterial flagellin by membrane and soluble orthologs of Toll-like receptor 5 in rainbow trout (Onchorhynchus mikiss).. J Biol Chem.

[39] Purcell MK, Smith KD, Hood L, Winton JR, Roach JC (2006). Conservation of Toll-Like Receptor Signaling Pathways in Teleost Fish.. Comp Biochem Physiol Part D Genomics Proteomics.

[40] Iliev DB, Roach JC, Mackenzie S, Planas JV, Goetz FW (2005). Endotoxin recognition: In fish or not in fish?. FEBS Letters.

[41] Sepulcre MP, Alcaraz-Perez F, Lopez-Munoz A, Roca FJ, Meseguer J (2009). Evolution of lipopolysaccharide (LPS) recognition and signaling: fish TLR4 does not recognize LPS and negatively regulates NF-kappaB activation.. J Immunol.

[42] Berczi I, Bertok L, Bereznai T (1966). Comparative studies on the toxicity of Escherichia coli lipopolysaccharide endotoxin in various animal species.. Can J Microbiol.

[43] Sepulcre MP, Lopez-Castejon G, Meseguer J, Mulero V (2007). The activation of gilthead seabream professional phagocytes by different PAMPs underlines the behavioural diversity of the main innate immune cells of bony fish.. Mol Immunol.

[44] Boltana S, Donate C, Goetz FW, MacKenzie S, Balasch JC (2009). Characterization and expression of NADPH oxidase in LPS-, poly(I:C)- and zymosan-stimulated trout (Oncorhynchus mykiss W.) macrophages.. Fish Shellfish Immunol.

[45] Iliev DB, Liarte CQ, MacKenzie S, Goetz FW (2005). Activation of rainbow trout (Oncorhynchus mykiss) mononuclear phagocytes by different pathogen associated molecular pattern (PAMP) bearing agents.. Mol Immunol.

[46] Dewitte-Orr SJ, Hsu HC, Bols NC (2007). Induction of homotypic aggregation in the rainbow trout macrophage-like cell line, RTS11.. Fish Shellfish Immunol.

[47] Flechner ER, Freudenthal PS, Kaplan G, Steinman RM (1988). Antigen-specific T lymphocytes efficiently cluster with dendritic cells in the human primary mixed-leukocyte reaction.. Cell Immunol.

[48] Romani N, Reider D, Heuer M, Ebner S, Kämpgen E (1996). Generation of mature dendritic cells from human blood An improved method with special regard to clinical applicability.. Journal of Immunological Methods.

[49] Kruse M, Rosorius O, Kratzer F, Bevec D, Kuhnt C (2000). Inhibition of Cd83 Cell Surface Expression during Dendritic Cell Maturation by Interference with Nuclear Export of Cd83 mRNA.. J Exp Med.

[50] Berchtold S, Muhl-Zurbes P, Heufler C, Winklehner P, Schuler G (1999). Cloning, recombinant expression and biochemical characterization of the murine CD83 molecule which is specifically upregulated during dendritic cell maturation.. FEBS Lett.

[51] Cain KD, Jones DR, Raison RL (2002). Antibody-antigen kinetics following immunization of rainbow trout (Oncorhynchus mykiss) with a T-cell dependent antigen.. Developmental & Comparative Immunology.

[52] Barr M, Mott K, Zwollo P (2011). Defining terminally differentiating B cell populations in rainbow trout immune tissues using the transcription factor XbpI.. Fish Shellfish Immunol.

[53] Utke K, Bergmann S, Lorenzen N, Köllner B, Ototake M (2007). Cell-mediated cytotoxicity in rainbow trout, Oncorhynchus mykiss, infected with viral haemorrhagic septicaemia virus.. Fish Shellfish Immunol.

[54] Wilson HL, Ni K, O'Neill HC (2000). Proliferation of dendritic cell progenitors in long term culture is not dependent on granulocyte macrophage-colony stimulating factor.. Exp Hematol.

[55] Kuwano Y, Prazma CM, Yazawa N, Watanabe R, Ishiura N (2007). CD83 influences cell-surface MHC class II expression on B cells and other antigen-presenting cells.. Int Immunol.

[56] Ohta Y, Landis E, Boulay T, Phillips RB, Collet B (2004). Homologs of CD83 from elasmobranch and teleost fish.. J Immunol.

[57] Donate C, Roher N, Balasch JC, Ribas L, Goetz FW (2007). CD83 expression in sea bream macrophages is a marker for the LPS-induced inflammatory response.. Fish Shellfish Immunol.

[58] Goetz FW, Iliev DB, McCauley LA, Liarte CQ, Tort LB (2004). Analysis of genes isolated from lipopolysaccharide-stimulated rainbow trout (Oncorhynchus mykiss) macrophages.. Mol Immunol.

[59] Martin SA, Zou J, Houlihan DF, Secombes CJ (2007). Directional responses following recombinant cytokine stimulation of rainbow trout (Oncorhynchus mykiss) RTS-11 macrophage cells as revealed by transcriptome profiling.. BMC Genomics.

[60] Haugarvoll E, Thorsen J, Laane M, Huang Q, Koppang EO (2006). Melanogenesis and evidence for melanosome transport to the plasma membrane in a CD83 teleost leukocyte cell line.. Pigment Cell Res.

[61] Roca FJ, Mulero I, Lopez-Munoz A, Sepulcre MP, Renshaw SA (2008). Evolution of the inflammatory response in vertebrates: fish TNF-alpha is a powerful activator of endothelial cells but hardly activates phagocytes.. J Immunol.

[62] Prazma CM, Yazawa N, Fujimoto Y, Fujimoto M, Tedder TF (2007). CD83 expression is a sensitive marker of activation required for B cell and CD4+ T cell longevity in vivo.. J Immunol.

[63] Kozlow EJ, Wilson GL, Fox CH, Kehrl JH (1993). Subtractive cDNA cloning of a novel member of the Ig gene superfamily expressed at high levels in activated B lymphocytes.. Blood.

[64] Yamashiro S, Wang J-M, Yang D, Gong W-H, Kamohara H (2000). Expression of CCR6 and CD83 by cytokine-activated human neutrophils.. Blood.

[65] Cao W, Lee SH, Lu J (2005). CD83 is preformed inside monocytes, macrophages and dendritic cells, but it is only stably expressed on activated dendritic cells.. Biochem J.

[66] Lechmann M, Shuman N, Wakeham A, Mak TW (2008). The CD83 reporter mouse elucidates the activity of the CD83 promoter in B, T, and dendritic cell populations in vivo.. Proc Natl Acad Sci U S A.

[67] Hartmann G, Weiner GJ, Krieg AM (1999). CpG DNA: a potent signal for growth, activation, and maturation of human dendritic cells.. Proc Natl Acad Sci U S A.

[68] Meloni S, Zarletti G, Benedetti S, Randelli E, Buonocore F (2006). Cellular activities during a mixed leucocyte reaction in the teleost sea bass Dicentrarchus labrax.. Fish Shellfish Immunol.

[69] Kaastrup P, Nielsen B, Horlyck V, Simonsen M (1988). Mixed lymphocyte reactions (MLR) in rainbow trout (Salmo gairdneri) sibling.. Dev Comp Immunol.

[70] Caspi RR, Avtalion RR (1984). The mixed leukocyte reaction (MLR) in carp: bidirectional and undirectional MLR responses.. Dev Comp Immunol.

[71] Sizemore RC, Miller NW, Cuchens MA, Lobb CJ, Clem LW (1984). Phylogeny of lymphocyte heterogeneity: the cellular requirements for in vitro mitogenic responses of channel catfish leukocytes.. J Immunol.

[72] Miller NW, Deuter A, Clem LW (1986). Phylogeny of lymphocyte heterogeneity: the cellular requirements for the mixed leucocyte reaction with channel catfish.. Immunology.

[73] Lund FE, Partida-Sanchez S, Lee BO, Kusser KL, Hartson L (2002). Lymphotoxin-alpha-deficient mice make delayed, but effective, T and B cell responses to influenza.. J Immunol.

[74] Hawiger D, Inaba K, Dorsett Y, Guo M, Mahnke K (2001). Dendritic cells induce peripheral T cell unresponsiveness under steady state conditions in vivo.. J Exp Med.

[75] Wells JW, Cowled CJ, Farzaneh F, Noble A (2008). Combined triggering of dendritic cell receptors results in synergistic activation and potent cytotoxic immunity.. J Immunol.

[76] Lemaitre B, Nicolas E, Michaut L, Reichhart J-M, Hoffmann JA (1996). The dorsoventral regulatory gene cassette spätzle/Toll/cactus controls the potent antifungal response in Drosophila adults.. Cell.

[77] Anderson KV, Jürgens G, Nüsslein-Volhard C (1985). Establishment of dorsal-ventral polarity in the Drosophila embryo: Genetic studies on the role of the Toll gene product.. Cell.

[78] Medzhitov R, Preston-Hurlburt P, Janeway CA (1997). A human homologue of the Drosophila Toll protein signals activation of adaptive immunity.. Nature.

