# Supplementary material for: Functional Identification of Dendritic Cells in the Teleost Model, Rainbow Trout (Oncorhynchus mykiss)
Source: PLoS One. 2012 Mar 12;7(3):e33196. doi: 10.1371/journal.pone.0033196 (PMC3299753; doi:10.1371/journal.pone.0033196)
Supplement: Table S1 — Primers List. Sequences and annealing temperatures of primers used for PCR amplification of immune-related and house-keeping (GPDH) genes from tDC cultures are listed, along with the expected sizes of amplicons for relevant primer pairs. Sources of primer sequences not listed in the references are indicated at the bottom of the table. (DOC) [file pone.0033196.s002.doc]

**Table S1.** **Primer List**

|  | Size (bp) | Forward primer | Reverse primer | Annealing temp. (°C) |
| --- | --- | --- | --- | --- |
| TLR-3 [1] | 538 | TGACAGAGCTTAACCTGGCT | AAGAACTTCCAGCATGGACA | 50 |
| TLR-5 | 214 | ACCTGTTCAATATCAGGGACGCCA | TGGGCAAAGGTCCTAATGCCTTCA | 63 |
| TLR-9 [2] | 247 | CCCCTTTGACTGCTCCTGTGACACTT | CATCCCAGCCGTAGAGGTGCCTGAGTAG | 63 |
| TLR-20 | 290 | GCGCTACTGCTATGATGCCTTTGT | TAAGGATGTCCCTTTGCTCCACCA | 63 |
| TLR-22 [3] | 150 | TGGACAATGACGCTCTTTTACC | GAGCTGATGGTTGCAATGAGG | 63 |
| TLR22L [3] | 148 | GGATGATGGGTCTCTCAGTCA | GAGCTGATGGTTGCAATGAGG | 63 |
| B7R | 405 | AAAGCATGACTGTGGACTACGGGA | AGTTGGCTGTCACACTGAGCTGTA | 50 |
| B7H1 | 619 | TTGTTCACAGTGGAGGTGGACAGT | ATACCTGGCTGATGGACCCTTTGT | 50 |
| B7H3 | 340 | ACACCACCCTCAACTGTTCCTTCA | AGGGTCACCAGAGGCTGGGAATA | 50 |
| B7H4 | 260 | TGTGACGTGACAGATGAACAGGGA | ACAGTCAGAGTGCTGTTCAACCAC | 63 |
| IL12p40 | 190 | GAACCCAGACGACGATGATT | GTTCAAACTCCAACCCTCCA | 50 |
| CXCR4 [4] | 198 | GTGCATGTGATCTACACCATC | GAGCTGTGGCAAACACTATGT | 63 |
| CCR7 [4] | 465 | TGTGACAAATCTGCCGTTAG | GCTCCTTGACGTTGGCGAACATG | 50 |
| MHCIIβ | 350 | AGCAGAGGAACATGTCGATGCCAA | AACTGTCTTGTCCAGTATGGCGCT | 50 |
| CD83 | 329 | TAACGGCTGTTGATAGCGGAAGGT | TATGGAACCCTGTCTCGACCAGTT | 50 |
| CD209 | 304 | CATCCTGCACAGCAAGAAACAGCA | TAGCCGAATGGCATCCATCTGACA | 50 |
| GPDH | 300 | GTCGTAAGACAGGATTGAGGC | CTGGGTCTCTCTCGTGGAAC | 50 |

1. Rodriguez MF, Wiens GD, Purcell MK, Palti Y (2005) Characterization of Toll-like receptor 3 gene in rainbow trout (Oncorhynchus mykiss). Immunogenetics 57: 510-519.

2. Ortega-Villaizan M, Chico V, Falco A, Perez L, Coll JM, et al. (2009) The rainbow trout TLR9 gene and its role in the immune responses elicited by a plasmid encoding the glycoprotein G of the viral haemorrhagic septicaemia rhabdovirus (VHSV). Mol Immunol 46: 1710-1717.

3. Rebl A, Siegl E, Kollner B, Fischer U, Seyfert HM (2007) Characterization of twin toll-like receptors from rainbow trout (Oncorhynchus mykiss): evolutionary relationship and induced expression by Aeromonas salmonicida salmonicida. Dev Comp Immunol 31: 499-510.

4. Daniels GD, Zou J, Charlemagne J, Partula S, Cunningham C, et al. (1999) Cloning of two chemokine receptor homologs (CXC-R4 and CC-R7) in rainbow trout Oncorhynchus mykiss. J Leukoc Biol 65: 684-690.
